# Slope of the power spectral density flattens at low frequencies (<150 Hz) with healthy aging but also steepens at higher frequency (>200 Hz) in human electroencephalogram

**DOI:** 10.1093/texcom/tgad011

**Published:** 2023-06-06

**Authors:** Srishty Aggarwal, Supratim Ray

**Affiliations:** Department of Physics, Indian Institute of Science, Bengaluru 560012, India; Centre for Neuroscience, Indian Institute of Science, Bengaluru 560012, India

**Keywords:** EEG, aperiodic activity, Alzheimer’s disease, alpha oscillation

## Abstract

The power spectral density (PSD) of the brain signals is characterized by two distinct features: oscillations, which are represented as distinct “bumps,” and broadband aperiodic activity, that reduces in power with increasing frequency and is characterized by the slope of the power falloff. Recent studies have shown a change in the slope of the aperiodic activity with healthy aging and mental disorders. However, these studies analyzed slopes over a limited frequency range (<100 Hz). To test whether the PSD slope is affected over a wider frequency range with aging and mental disorder, we analyzed the slope till 800 Hz in electroencephalogram data recorded from elderly subjects (>49 years) who were healthy (*n* = 217) or had mild cognitive impairment (MCI; *n* = 11) or Alzheimer’s Disease (AD; *n* = 5). Although the slope reduced up to ~ 150 Hz with healthy aging (as shown previously), surprisingly, at higher frequencies (>200 Hz), it increased with age. These results were observed in all electrodes, for both eyes open and eyes closed conditions, and for different reference schemes. However, slopes were not significantly different in MCI/AD subjects compared with healthy controls. Overall, our results constrain the biophysical mechanisms that are reflected in the PSD slopes in healthy and pathological aging.

## Introduction

Neural signals such as electroencephalography (EEG), magnetoencephalography (MEG), electrocorticography (ECoG), and local field potential (LFP) provide critical insights into the physiological processes underlying key aspects of human cognition and neurodevelopment. These signals often reveal oscillations at different frequencies, which are represented as “bumps” in the power spectral density (PSD), and have been extensively studied as an objective measure for cognitive phenotyping (Klimesch 1999; Schutter and Knyazev, 2012), biomarkers for age (Başar 2013; Murty et al. 2020), as well as neurological disorders (Newson and Thiagarajan 2018; Murty et al. 2021). In addition, the aperiodic background activity in the signals, often known as the “1/f component” or “scale-free activity,” is characterized by the slope or exponent of the PSD (on a log–log scale) in a specified frequency band (He 2014).

The PSD slope has garnered interest in recent years, as has been shown to be one of the key features of signal variability (Ribeiro and Castelo-Branco 2022) and has been related to N900 lexical prediction (Dave et al. 2018), working memory (Donoghue et al. 2020b), grammar learning (Cross et al. 2022), sleep changes (Bódizs et al. 2021), anesthesia (Kreuzer et al. 2020), and EEG fingerprinting (Demuru and Fraschini 2020). The slope has been suggested to depend on multiple factors that include self–organized criticality (Mandelbrot 2013), excitation-inhibition balance (Freeman and Zhai 2009; Gao et al. 2017), dendritic response to an input (Voytek and Knight 2015), tissue properties (Bédard et al. 2006a), temporal dynamics of the synaptic processes triggered by Poisson spiking (Milstein et al. 2009), and ionic diffusion processes across the extracellular membrane (Bédard and Destexhe 2009). More recently, it has been linked to the “neural noise” hypothesis ((Voytek et al. 2015); more details in the Discussion section). The slope also depends on the type of signal. For example, slope for ECoG is much steeper than LFP between 20 and 100 Hz but becomes comparable between 200 and 400 Hz (Dubey and Ray 2019).

Change in aperiodic activity is suggested to be a plausible biomarker for some neurological and psychiatric diseases like schizophrenia (Molina et al. 2020), attention deficit hyperactivity disorder (ADHD; Robertson et al. 2019; Ostlund et al. 2021), and Fragile X Syndrome (Wilkinson and Nelson 2021), although the aperiodic activity was unchanged in other diseases such as Alzheimer’s disease (AD) (Benwell et al. 2020; Springer et al. 2022). Recently, reduction in the slope with increasing age has been observed across several studies under different task paradigms (Voytek et al. 2015; Dave et al. 2018; Tran et al. 2020) or even in resting-state conditions (Thuwal et al. 2021; Hill et al. 2022; Merkin et al. 2023). However, slope analysis in these studies has been restricted to frequency ranges below 125 Hz, with most of them only up to 50 Hz. Indeed, very few reports have studied the PSD slopes in EEG at frequencies above 150 Hz (Dehghani et al. 2010), but the effect of aging or mental disorder on the PSD slopes at these frequencies is unknown.

Higher frequencies have generally been ignored because they have low absolute power and therefore could be more susceptible to instrumentation noise or other artifacts related to muscle activity. However, the definitions of “noise” and “signal” are rather nuanced (see (Uddin 2020), for a comprehensive discussion)—“1/f noise” could arise due to signal variability organized across various spatial and temporal scales, and therefore the higher frequencies could reflect important information about neural processes occurring over fast timescales, such as synaptic neurotransmitter diffusion time (Sabatini and Regehr 1996). For example, power in the “high-gamma” range (>80 Hz) has been shown to accurately reflect multi-unit firing in the LFP (Ray and Maunsell 2011), with a very high correlation between 250 and 500 Hz. Similarly, the oscillations in high-frequency range (HFR, 300–500 Hz) have been shown to exhibit age-dependent changes (Nakano and Hashimoto 1999, 2000). This prompted us to investigate the aperiodic activity in these HFRs.

In this study, we explored aperiodic activity in the EEG activity up to 800 Hz in healthy elderly subjects aged 50–88 years. We analyzed the slopes in both eyes open and eyes closed states. Eyes closed state enabled us to minimize potential electromyography (EMG) artifacts that could be present in the eyes open state. We further examined the dependence of slopes on the reference schemes. Finally, we compared the slopes in subjects with mild cognitive impairment (MCI) and early AD with their age and gender matched healthy controls.

## Materials and methods

The details of experimental setup and data collection have been explained in detail in previous studies (Murty et al. 2020, 2021; Kumar et al. 2022). The data are already published; here, we carried out further analysis of the slope of the PSD which was computed over a much wider frequency range than before. In addition, we have used a novel dataset involving “eyes closed” condition, which was not used previously. We summarize the details below in brief.

### Dataset

We used the EEG dataset collected from 257 human subjects (148 males and 109 females) aged 50–88 years (Murty et al. 2020, 2021) under Tata Longitudinal Study of Aging (TLSA) who were recruited from the urban communities of Bengaluru, Karnataka, India. They were clinically diagnosed by psychiatrists, psychologists and neurologists at National Institute of Mental Health and Neurosciences (NIMHANS), and M.S. Ramaiah Hospital, Bengaluru as cognitively healthy (*n* = 236), or with MCI (*n* = 15) or AD (*n* = 6), using a combination of tests such as the Clinical Dementia Rating scale (CDR), Addenbrook’s Cognitive Examination-III (ACE-III), and Hindi Mental State Examination (HMSE). Diagnosis of all MCI/AD subjects was reviewed by a panel of four experts who reclassified two MCI subjects as healthy. Data from these two subjects were not taken for analyses. Further, 11 (10 healthy and 1 MCI) subjects were discarded due to noise (See Artifact Rejection subsection). One AD patient was further discarded as there was no age and gender matched control. We further discarded another 10 subjects (9 healthy and 1 MCI) for whom data was collected using 32 channels, leading to the usable 64-channel data of 233 subjects (217 healthy, 11 MCI and 5 AD). The eyes closed data (see the next subsection) was not collected from 16 subjects (13 healthy, 2 MCI, and 1 AD), leading to a slightly smaller dataset for the eyes closed condition.

Informed consent was obtained from the participants of the study and monetary compensation was provided. All the procedures were approved by the Institute Human Ethics Committees of Indian Institute of Science, NIMHANS, and M.S. Ramaiah Hospital, Bengaluru.

### Experimental settings and behavioral task

Briefly, EEG was recorded from 64-channel active electrodes (actiCap) using BrainAmp DC EEG acquisition system (Brain Products GMbH). The electrodes were placed according to the international 10–10 system, referenced online at FCz. Raw signals were filtered online between 0.016 Hz (first-order filter) and 1 kHz (fifth-order Butterworth filter), sampled at 2.5 kHz, and digitized at a 16-bit resolution (0.1 μV/bit). The subjects were asked to sit in a dark room in front of a gamma corrected LCD monitor (BenQ XL2411; dimensions: 20.92 × 11.77 inches; resolution: 1,280 × 720 pixels; refresh rate: 100 Hz) with their heads supported by a chin rest. It was placed at (mean ± SD) 58 ± 0.7 cm from the subjects (range: 54.9–61.0 cm) and subtended 52${}^{\circ}$ × 30${}^{\circ}$ of visual field for full screen gratings. Eye position was monitored using EyeLink 1000 (SR Research Ltd), sampled at 500 Hz.

In the beginning of the experiment, the resting state EEG for the eyes closed condition was collected for 1–2 min. Then, the subjects performed a passive visual fixation task, including the full screen grating stimuli. The task consisted of a single session that lasted for ~ 20 min, divided in 2–3 blocks with 3–5 min breaks in between, according to subjects’ comfort. Every trial started with the onset of a fixation spot (0.1${}^{\circ}$) shown at the centre of the screen, on which they were instructed to fixate. After an initial blank period of 1,000 ms, two to three full screen grating stimuli were presented for 800 ms with an interstimulus interval of 700 ms using a customized software running on MAC OS. The stimuli were full contrast sinusoidal luminance achromatic gratings with either of the three spatial frequencies (one, two, and four cycles per degree (cpd)) and four orientations (0${}^{\circ}$, 45${}^{\circ}$, 90${}^{\circ}$, and 135${}^{\circ}$). Our analyses were restricted to 500 ms of the interstimulus period before the onset of the stimulus, referred as the baseline period in the previous studies (Murty et al. 2020, 2021). We refer to it as “eyes open” condition.

### Artifact rejection

For eyes open data, we used the artifact rejection framework as described in (Murty et al. 2020, 2021; Murty and Ray 2022) and the steps are summarized here:

(a) Eye-blinks or change in eye position outside a 5${}^{\circ}$ fixation window during −0.5 to 0.75 s from stimulus onset were noted as fixation breaks and removed offline. This led to a rejection of 14.6 ± 2.8% (mean ± SD) repeats.
(b) All the electrodes with impedance > 25 k$ \Omega $ were rejected. Impedance of final set of electrodes was (mean $ \pm $ SD) 5.48 $ \pm $ 1.83 k$ \Omega $
(c) In the remaining electrodes, outliers were detected as repeats with deviation from the mean signal in (i) time or (ii) frequency domains by more than 6 standard deviations, and subsequently electrodes with more than 30% outliers were discarded.
(d) Further, repeats that were deemed bad in the visual electrodes (P3, P1, P2, PO3, POz, PO4, O1, Oz, and O2) or in more than 10% of the other electrodes were considered bad, eventually yielding a set of common bad repeats for each subject. Overall, this led us to reject (mean ± SD) 15.58 ± 5.48% repeats.
(e) We computed slopes for the power spectrum between 56 and 84 Hz for each unipolar electrode and rejected electrodes whose slopes were less than 0. Overall, it led to a rejection of (19.45 $\pm\: 14.64\%$) electrodes.
(f) We found that a small fraction of electrodes/stimulus repeats had either very small or very large signals that were not getting discarded using the pipeline above. Therefore, in addition to the artifact detection pipeline that was used in the previous studies, for each electrode, we computed the root mean square value (RMS) of the time series for all the remaining trials (after removing common bad repeats using the above criteria), and declared repeats having RMS values lower than 1.25 μV or higher than 35 μV as new outliers. We then rejected the electrodes that had more than 30% new outliers. Further, any new outlier if it belonged to the visual electrodes or was common in more than 10% of other electrodes was considered a bad repeat and appended to the existing list of bad repeats. This led to rejection of additional 1.24 $\pm$ 1.72 electrodes and 0.16 $\pm$ 1.05% repeats. This condition was added mainly to improve PSD plots unlike the previous studies (Murty et al. 2020, 2021; Kumar et al. 2022) that dealt mainly with change in power. The main results remained similar even without applying this new criterion.
(g) We further discarded the blocks that did not have at least a single good unipolar electrode in the left visual anterolateral (P3, P1, PO3, O1), right visual anterolateral (P2, P4, PO4, O2), and posteromedial (POz, Oz) electrode groups. We then pooled data across all good blocks for every subject separately for the final analysis. Those subjects who did not have any analyzable blocks (10/237 healthy and 1/15 MCI) were discarded for further analysis.

The eyes closed EEG data were segmented into non-overlapping 2-s epochs, resulting in a higher resolution of 0.5 Hz. Each segment was then treated as a “stimulus repeat” and subjected to the same artifact rejection pipeline as the eyes open data, with minor changes as described below. We only considered subjects who were deemed good for the eyes open condition. Then, we started with step (b) above, yielding the average impedance of 5.47 $\pm$ 1.77 k$\Omega$. We then applied the RMS criteria in place of deviation from mean signal in time domain in (c). Since, we were applying RMS criteria at an initial step on the raw data containing the outliers, we had to increase the lower cut off to 2.5 μV and keep the upper cut off same. We repeated the remaining steps for artifact rejection done for eyes open from (c) (ii)—(e), i.e. detection of outliers from standard deviation in frequency domain to rejection of electrodes with slopes less than 0 as described above. It led to the exclusion of (3.11 $\pm\: 4.87\%$) repeats and (18.86 $\pm$ 13.10%) electrodes. No additional subject was rejected when we applied the criteria in (g) for eyes closed data.

After discarding all the bad repeats, (293.42 $\pm$ 67.22) and (39.35 $\pm$ 7.75) repeats were available for eyes open and eyes closed conditions, respectively.

### E‌EG data analysis

Our primary emphasis was to characterize slope of the aperiodic activity in the PSD as a function of age within the elderly population (>49 years), for which we divided these subjects into two groups: 50–64 (Mid) and > 64 years (Old), as depicted in Table 1. We also compared the slopes in subjects with AD/MCI (termed “cases”) with their healthy, age and gender matched controls. As in our previous studies (Murty et al. 2021; Kumar and Ray 2023), for each case, we averaged the relevant metrics for all age ($\pm$ 1 year) and gender matched controls to yield a single control data point for each case, yielding 16 (13) pairs for eyes open (eyes closed) analyses (Table 1).

**Table 1:** Number of subjects in each group for the eyes open and eyes closed conditions. The numbers in parenthesis indicate the male (M) and female (F) subjects separately.

	Mid (50–64 years)	Old (>64 years)	MCI	AD
Eyes open	90 (40 M, 50 F)	127 (79 M, 48 F)	11 (9 M, 2 F)	5 (3 M, 2 F)
Eyes closed	82 (37 M, 45 F)	122 (74 M, 48 F)	9 (8 M, 1 F)	4 (2 M, 2 F)

All the data analyses were done using custom codes written in MATLAB (MathWorks. Inc; RRID:SCR_001622). Similar to the previous studies (Murty et al. 2020, 2021; Kumar et al. 2022) on this dataset, we chose [−500 0] ms as the eyes open period, yielding a resolution of 2 Hz. The analyses were performed using unipolar reference scheme, unless otherwise specified. Power spectrum was obtained using the multi-taper method with a single taper using the Chronux Toolbox ((Bokil et al. 2010), RRID:SCR_005547) for individual trials and then averaged across the trials for each electrode, which reduces the noise in the spectral estimator (Jarvis and Mitra 2001).

#### Slope analysis

The slope of the 1/*f* aperiodic component of the trial-averaged PSD for each electrode was computed using the Matlab wrapper for the Fitting Oscillations and One Over f (FOOOF) toolbox (Donoghue et al. 2020b). In FOOOF, the power spectrum *P*(*f*) for frequency *f* is modeled as a combination of aperiodic *AP(f)* and oscillatory components and can be expressed as

$$ P(f)= AP(f)+{\sum}_n{G}_n(f). $$

The *AP(f)* is given by

$$ AP(f)={10}^b{f}^{-\chi }, $$

where $\chi$ is the exponent or “slope” and *b* is the offset. Each oscillatory contribution ${G}_n(f)$ is modeled as a Gaussian peak:

$$ {G}_n(f)={a}_n\exp \left[-\frac{{\left(f-{\mu}_n\right)}^2}{2{\sigma}_n^2}\right], $$

with ${a}_n$ as the peak height, ${\mu}_n$ as the centre frequency and ${\sigma}_n$ as the width of each component. The settings chosen for FOOOF model parameters were: peak width limits: [4,8] for eyes open and [1,8] for eyes closed (different values were used to account for differences in frequency resolution in the two datasets); maximum number of peaks: 5; minimum peak height: 0.2; peak threshold: 2.0; and aperiodic mode: “fixed.”

In our data, peaks in the PSD were only evident in the alpha range (8–12 Hz) and line noise (50 Hz) and its harmonics. By taking a frequency range that did not include these oscillatory frequencies (or by not considering these frequencies for analysis), the slope can also be estimated by simply fitting a straight line to log(*P*(*f*)) vs log(*f*) plot, as done in a previous study (Shirhatti et al. 2016). We employed least-squares minimization using the program *fminsearch* in Matlab to obtain the slope, which gave similar results to the slopes estimated using FOOOF. This was done in order to verify the results that were obtained using FOOOF and to also validate the FOOOF fitting procedure for higher frequency ranges.

Since the slope does not remain constant throughout the full frequency range (Shirhatti et al. 2016), we fitted the PSD segments in frequency ranges from 4 to 1,000 Hz in steps of 20 Hz with the centre frequency between 40 and 960 Hz, having the frequency width of 100 Hz. The PSD segments with the lowest and highest centre frequencies had slightly smaller frequency range width owing to frequency limit of 4–1,000 Hz. We also fitted a single slope in the low-frequency range (LFR) (64–140 Hz) and the HFR (230–430 Hz) to verify that the trend in slope is not attributable to a particular frequency range width and length of PSD segments. Note that FOOOF has a limitation that if the frequency range to be fitted has peaks at its ends, the evaluated slope is inappropriate (Gerster et al. 2022). To overcome this limitation, we declared $\pm\: 4\ \text{Hz}$ around the peaks corresponding to the line noise at 50 Hz and its harmonics as “noise peaks,” and avoided frequency ranges for which these noise peaks lay near their end points. We also did not use slopes that were less than 0.01, which may be due to poor fitting.

#### Linear regression

The slope was modeled to vary linearly with age. We used *fitlm* function in Matlab that generates the model parameters $\beta 1,\beta 2$ corresponding to $y=\beta 1+\beta 2\ast x,$ R-squared (${R}^2$) and *P*-value using *t*-statistics.

#### Electrode grouping

Scalp maps were generated using the topoplot function of EEGLAB toolbox ((Delorme and Makeig 2004), RRID:SCR_007292) with standard *Acticap 64* unipolar montage of the channels. We divided the electrodes into five groups as occipital (O1, Oz, O2, PO3, PO4, PO7, PO8, PO9, PO10, POz), centro-parietal (CP1, CP2, CP3, CP4, CP5, CP6, CPz, P1, P2, P3, P4, P5, P6, P7, P8, Pz), fronto-central (FC1, FC2, FC3, FC4, FC5, FC6, C1, C2, C3, C4, C5, C6, Cz), frontal (Fp1, Fp2, F1, F2, F3, F4, F5, F6, F7, F8, Fz, AF3, AF4, AF7, AF8), and temporal (T7, T8, TP7, TP8, TP9, TP10, FT7, FT8, FT9, FT10). FCz and Fpz were used as the reference and the ground, respectively.

In our previous studies (Murty et al. 2020, 2021; Kumar et al. 2022), power analysis was done on electrodes for which strong gamma power was observed (P3, P1, P2, PO3, POz, PO4, O1, Oz and O2), which were termed as “high priority” electrodes. We therefore started with the slope analysis on this group and later extended to all the electrode groups.

### Statistical analysis

The statistical analysis was done for slope comparison. Kruskal–Wallis (K-W) test, Wilcoxon Rank Sum (WRS) test were used to compare the medians across groups to be consistent with previous studies (Murty et al. 2020, 2021). The standard error of median (SEM) was computed after bootstrapping over 10,000 iterations. Similar results were obtained when we used the mean instead of the median and ANOVA instead of K-W test (data not shown).

To remove false alarms created by low *p*-values at certain frequencies, we applied cluster correction (Cohen 2014). We used *bwconncomp* function of Image Processing toolbox in Matlab to identify the frequency clusters having *p*-values less than 0.05 and 0.01. We called a cluster significant if at least three consecutive frequency bins were significant. For correction for multiple comparisons across electrodes in topoplots, we used the false discovery rate (FDR) using the Benjamini–Hochberg procedure for independent tests (Benjamini and Hochberg 1995).

## Results

We examined EEG data for 217 healthy adults aged between 50 and 88 years, grouped as Mid (50–64 years; *n* = 90) and Old (>64 years; *n* = 127). The subjects sat with their eyes closed for 1–2 min before performing a fixation task in which full-screen gratings were presented for 800 ms with an inter-stimulus interval of 700 ms. We computed the PSD of 500 ms segments of data during interstimulus period to get the “eyes open” condition, yielding a frequency resolution of 2 Hz. To overcome the plausible electromyography (EMG) artifacts in eyes open state, we also analyzed the eyes closed data with segments of 2 s each, which yielded a higher resolution of 0.5 Hz. We computed the slopes using Matlab wrapper for FOOOF toolbox (Donoghue et al. 2020b), in steps of 20 Hz between 4 and 1,000 Hz with centre frequencies from 40 to 960 Hz by taking PSD segments of $\pm\: 50$ Hz around each centre frequency. The first and the last PSD segments were slightly smaller owing to frequency limit of 4–1,000 Hz. Slopes less than 0.01 were not considered for analysis (see Materials and Methods). We have shown the PSDs and slopes up to 800 Hz to avoid the influence of filter roll off caused by a fifth order Butterworth filter at 1,000 Hz.

### Slopes vary differently with age in low- and high-frequency regimes

**Fig. 1:**
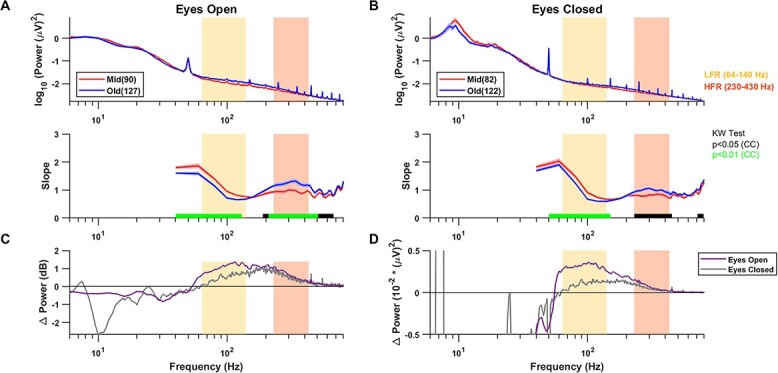
(A) PSDs (top) and slopes (bottom) for the two age groups in high priority electrodes in eyes open state. Solid traces represent the median for mid and old age groups respectively and shaded region around them indicates $\pm$ SEM across subjects, computed after bootstrapping over 10,000 iterations. The numbers in legend in the top panel represent the subjects in the respective age groups. Horizontal bars at the abscissa in the bottom panel represent significance of differences of slopes between mid and old (black: *P* < 0.05 and green: *P* < 0.01, K-W test, cluster corrected (CC)). Yellow and orange boxes represent the LFR (64–140 Hz) and the HFR (230–430 Hz), respectively. (B) Same as (A) in the eyes closed condition. (C) The median change in power between mid and old in decibels (dB) for the eyes open (purple) and eyes closed (gray) conditions. (D) Median change in absolute power between mid and old for these two conditions.

Figure 1(A) shows the median PSDs and slopes with frequencies for the mid and old groups for the eyes open condition for the “high-priority” electrode group (see Methods for details). Like the slope variation in LFP data in monkeys (Shirhatti et al. 2016), for both age groups, slope was high initially due to the presence of oscillatory activities and decreased beyond ~ 60 Hz until ~ 140 Hz. It then increased again after a “knee” at ~ 175 Hz (see bottom plot in Fig. 1A). The PSDs flattened out for old subjects than mid aged subjects from ~ 50 Hz until ~ 140 Hz, resulting in lower slopes for the former in the LFR 64–140 Hz, as highlighted by yellow boxes. This is in consensus with the results reported previously (Voytek et al. 2015; Merkin et al. 2023). However, beyond ~ 200 Hz, the trend reversed and the PSDs for old subjects became steeper, resulting in steeper slopes beyond ~ 200 Hz up to ~ 700 Hz. The HFR at 230–430 Hz, marked in orange, highlights the prominent region of higher slopes for the old group.

Since the frequency resolution for the eyes open condition was 2 Hz, and there could be some EMG activity related to maintenance of fixation, we also studied the slopes when eyes were closed for which longer segments of data were used to get a frequency resolution of 0.5 Hz (Fig. 1B). This condition revealed a more prominent alpha peak as compared with the eyes open condition, and also showed a well-known slowing of the alpha wave with aging (Scally et al. 2018; Merkin et al. 2023), with the centre frequency reducing from 9.72 $\pm$ 0.11 in mid to 9.41 $\pm$ 0.09 in old-aged group (*P* = 0.008, WRS test). Importantly, the flattening of slope in LFR and steepening in HFR with age was also observed in this dataset (Fig. 1B, bottom panel).

To better quantify the factors that could lead to the change in PSDs, we first compared the change in PSD between mid and old groups by subtracting the log PSD plots for the two conditions shown in Fig. 1(A) and (B) (and multiplying by 10 to get units of decibels; Fig. 1C). As power is subtracted on a log scale, it is simply the log of the ratio of powers, and therefore, represents the scaling factor that must be applied on the PSD for the mid group to obtain the PSD for the old group. This log ratio was negative (scaling factor < 1) below ~ 50 Hz, and subsequently became positive with a peak between 100 and 200 Hz. This ratio was smaller for the eyes closed condition in the LFR but comparable in the HFR.

We also considered an additive noise hypothesis in which noise was added to the signal obtained from the mid group to get the signal for the old group. If the noise at any frequency is independent of the signal, the power is additive, such that the power of the required noise can be obtained by simply subtracting the PSDs but on a linear (rather than log as done in Fig. 1C) scale (Fig. 1D). This also yielded a shallow “bump” between 50 and 500 Hz peaking at around ~ 100 Hz (values below ~ 50 Hz are negative due to a reduction of alpha and beta power with aging). As in Fig. 1(C), noise power was lower in LFR for eyes closed than the eyes open condition, potentially reflecting some contribution of EMG which is prominent between 40 and 200 Hz (Muthukumaraswamy 2013; McManus et al. 2020). However, this “noise” was comparable in the HFR range for the two conditions.

### Age variation of slope in LFR and HFR is observed in all electrodes

**Fig. 2:**
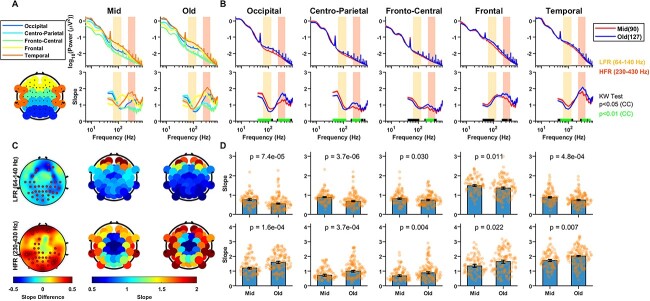
Variation of PSDs and slopes with frequency across the two age groups for different electrodes for the eyes open state. (A) PSDs and slopes across different electrode groups for mid and old age groups. The top plot in the left bottom panel highlights the electrodes chosen for each electrode group. (B) PSDs and slopes between the age groups for each electrode group separately. Solid traces represent the median and shaded region around them indicates $\pm$ SEM across subjects, computed after bootstrapping over 10,000 iterations. The numbers in legend in the top panel represent the subjects in the respective age groups. Colored bars at the abscissa in the bottom panel represent significance of differences of slopes between mid and old (black: *P* < 0.05 and green: *P* < 0.01, K-W test (CC)). Yellow and orange boxes represent the LFR (64–140 Hz) and the HFR (230–430 Hz), respectively, in (A) and (B). (C) Topoplot of the difference in slopes between old and mid age groups (left column), computed in LFR (top row) and HFR (bottom row), and the raw slope values in the mid (central column) and old (right column) age groups. Electrodes with significant difference (*P* < 0.05, WRS test, FDR corrected) in slopes are highlighted in red in the left panel. (D) Bar plots showing median slopes for each electrode group for mid- and old-aged people for LFR and HFR, with error bars representing standard error of median (obtained using bootstrapping). The WRS test *p*-values are indicated at the top of the bar plots and the dots represent the slope for each subject.

Next, we compared the slopes for different sensors, for which the electrodes were grouped in five categories as mentioned in Materials and Methods. Figure 2(A) shows the PSDs and slopes in all the electrode groups for the eyes open condition. Interestingly, the PSD slopes varied considerably depending on electrode location over the entire frequency range. Figure 2(C) shows the fitted slopes in LFR and HFR. In particular, in HFR, the slopes increased radially from the central area.

**Fig. 3:**
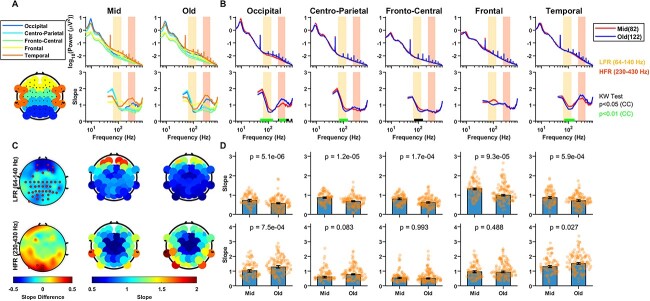
Variation of PSDs and slopes with frequency across the two age groups in various areas of brain for the eyes closed state. Same as Fig. 2 but for the eyes closed condition.

In spite of the differences in absolute slope values, the difference in slope between old and mid aged groups showed consistent trends (Fig. 2B). The difference in slopes between old and mid groups in LFR (Fig. 2C; top plot) and HFR (Fig. 2C; bottom plot) were consistently negative and positive, respectively. So, the posterior–anterior shift in aging (PASA), commonly observed in functional neuroimaging studies of aging and characterized by age-related reduction in occipital activity alongside increase in frontal activity (Mccarthy et al. 2014), was not observed in aperiodic activity in the present dataset. The electrodes for which the differences were significant (*P* < 0.05, WRS test, false discovery rate (FDR) corrected; red dots in Fig. 2C) were more concentrated in the posterior and occipital electrodes. Figure 2(D) shows the median value of the slopes in two age groups in LFR (top) and HFR (bottom), which were significantly different in all electrode groups (*p*-values are shown in the plots). The results were similar when the analysis was restricted to only males or females (data not shown). Similar analyses were performed for eyes closed state as shown in Fig. 3. The results in eyes closed state were similar to the eyes open state, although the differences in slopes between the age groups (Fig. 3C) were less prominent and only significant in few electrodes in the occipital area for HFR (Figs. 3C and D).

The median goodness of fit (*R*^2^) for all electrodes across the frequency range in these two conditions is shown in Supplementary Fig. 1. *R*^2^ was relatively high at frequencies in LFR and gradually decreased at higher frequencies, potentially due to lower absolute power. *R*^2^ was also lower for the eye-closed condition as compared with eyes open, due to lower number of trials that were used to obtain the PSD in the former case. Interestingly, in LFR, it was significantly higher for mid-aged group as compared with old-aged group. To rule out the possibility of differences in *R*^2^ affecting our results, we sub-sampled from each population to match the distribution of *R*^2^ values and still found that the slopes remained different between mid and old groups. Further, in HFR, *R*^2^ values were not significantly different between the two age groups, providing additional confidence in the slope results in this frequency range.

While reporting aperiodic activity, previous studies have included the variation of exponent as well as offset. In general, the rotation of PSD changes the exponent and offset together leading to strong correlation between these two parameters, as reported previously (Hill et al. 2022; Merkin et al. 2023). Changes in offset (while keeping the exponent same) can be observed in task-related studies that corresponds to broadband change in PSD (Voytek and Knight 2015). However, we observed similar changes in offset with age in LFR and HFR as the exponent (Supplementary Fig. 2), and, hence, have focused only on the exponent variation.

### Slope variation across electrodes is independent of the reference scheme

**Fig. 4:**
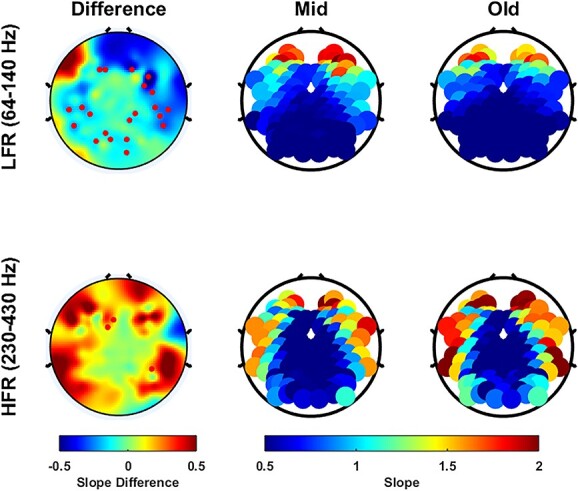
Same as Fig. 2(C) but for the bipolar reference scheme.

In HFR, the slopes were lower for the central electrodes and increased across frontal, temporal and occipital areas (Fig. 2(C) and Fig. 3(C); bottom panels). In our recording setup, the reference and ground electrodes were near the centre and, therefore, the radial increase in slopes in HFR could be due to an increase with the distance of the electrode from the reference electrode. To test the dependence of HFR slope on the reference scheme, we re-referenced all signals using a bipolar referencing scheme in which each electrode was referenced with respect to a neighboring electrode to yield a virtual bipolar electrode at the mid-point of the two electrodes, yielding 112 bipolar-referenced electrodes (for more details, see Murty et al. 2020). Figure 4 shows the same results as Fig. 2(C) after computing the slopes for the bipolar signals. The results after bipolar referencing remained similar, although less significant, thus repudiating the variation across electrodes as an artifact, although the absolute slopes for bipolar reference scheme were slightly lower than unipolar reference scheme, as also shown previously (Shirhatti et al. 2016).

### Slopes do not vary between AD/MCI patients and their controls

**Fig. 5:**
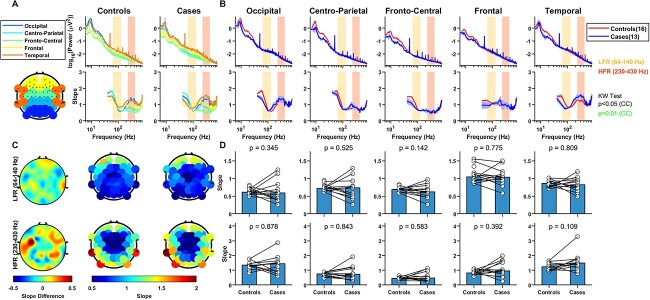
Comparison of PSDs and slopes with frequency between controls and cases (AD/MCI) for the eyes closed state. (A)–(C) are similar to Figs. 2(A–C). Since there were several controls per case, median PSDs/slopes across all controls for each case subject was used to match the numbers of cases and controls. (D) The bar plots show the median slope for each electrode group in LFR and HFR across controls and cases. Individual case and control pair are shown as connected white dots. The WRS test *p*-values are indicated on the top of the bar plots.

We had 107 healthy subjects for the eyes open and 100 for the eyes closed conditions who were age and gender matched with the AD/MCI subjects (6–19 controls per case subject; see Murty et al. 2021, for details). To make the comparison more balanced, similar to our previous studies (Murty et al. 2021; Kumar and Ray 2023), we computed the median PSDs/slopes across all controls for a given case subject to match the number of cases and controls and subsequently performed pairwise analysis. Figure 5 shows the results for the eyes closed condition (*n* = 13). The PSDs revealed a significant reduction in beta power (15–35 Hz) in cases compared with controls in all the electrode groups as reported in other studies (Ranasinghe et al. 2022). For example, it reduced from 12.14 $\pm$ 1.56 and 11.18 $\pm\: 1.32\ \mu{V}^2$ in controls to 5.83 $\pm$ 1.16 and 5.78 $\pm$ 1.24 $\mu{V}^2$ in cases in occipital and temporal electrodes (*P* = 0.0048 and 0.0066, WRS test), respectively. This reduction was observed in the eyes open condition also (*n* = 16), although it did not reach significance (data not shown). However, there was no significant change in slopes between controls and cases in either LFR or HFR in either eyes open and eyes closed conditions (eyes closed: Fig. 5B–D), consistent with previous studies which also did not find any changes in the PSD slope with MCI/AD (Benwell et al. 2020; Springer et al. 2022).

### Variation in slope with age is confirmed using regression analysis

**Fig. 6:**
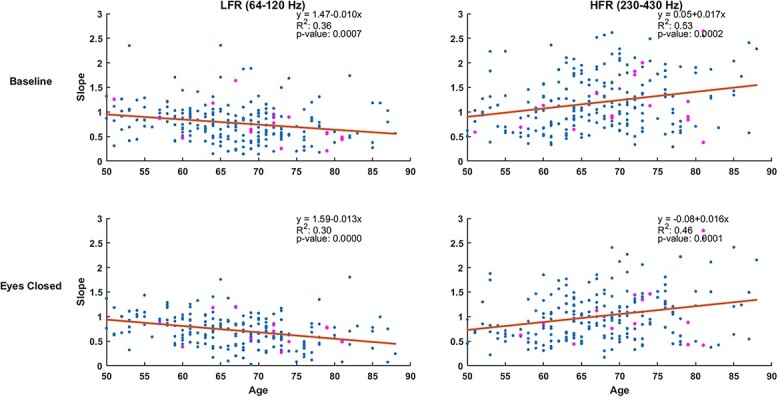
Scatter plots showing the slopes in high priority electrodes for all subjects in LFR and HFR for baseline and eyes closed conditions. Blue dots represent healthy subjects while MCI and AD subjects are indicated using pink dots. The regression line is in black and the corresponding parameters obtained using regression analysis are shown in top right side of each subplot.

To confirm the variation in slope across subjects without grouping them in predefined groups, we regressed the slopes in the high priority electrode group with age (see Material and Methods for details) for the two frequency ranges and the two conditions, as shown in Fig. 6. Consistent with previous results, we observed a negative relationship in LFR (slope = −0.010 for eyes open and − 0.013 for eyes closed) and a positive relationship in HFR (slope = 0.017 for eyes open and 0.016 for eyes closed). Interestingly, slope magnitude in HFR was higher than LFR, indicating a faster change of slope with age in HFR. Also, ${R}^2$ was higher in HFR (0.53 eyes open, 0.46 eyes closed) than LFR (0.36 eyes open, 0.30 eyes closed). All the changes were significant (LFR: *P* = 0.0007 eyes open, *P* = 0.0001 eyes closed; HFR: *P* = 0.0002 eyes open, *P* = 0.0001 eyes closed). There was nearly equal distribution of AD/MCI cases, shown as pink dots, around the regression line indicating the indifference of their slopes with healthy subjects in both LFR and HFR for the eyes open as well as eyes closed conditions.

We used this simple model containing the linear regression analysis to highlight the age-related decrease and increase in slopes in LFR and HFR respectively. However, as shown in Fig. 6, the inter-subject data were noisy and the linear model could be too simplistic, as the characteristics of the slope of the ongoing activity in brain are dynamic. One possible mechanism for this dynamic change is a phenomenon called self-organized criticality (Bak et al. 1987), which may give rise to nonlinearity in the association of age with slope of the 1/*f* component. Indeed, origin of “1/f noise” may lie in tissue properties (Bédard et al. 2006a), which could change in a non-linear fashion with age. Nevertheless, the linear model is sufficient to validate the observed pattern in age-related changes in slopes, as shown by age-wise grouping the subjects in previous figures.

## Discussion

We investigated age and MCI/AD-related changes in the EEG aperiodic activity by studying the variation in the slope of the PSD over a broad frequency range (up to 800 Hz). In a task where subjects maintained fixation on the screen while visual stimuli were presented (Murty et al. 2020, 2021), we computed the PSDs during the inter-stimulus interval period. Consistent with previous studies, PSD slope flattened with age up to ~ 150 Hz. However, the slope showed a surprising increase with age at frequencies beyond 200 Hz. This age-related distinct behavior of slopes at LFRs and HFRs was observed in all sensors, although, the slopes varied with the sensor location. These results were confirmed using another dataset from the same subjects when they sat quietly with their eyes closed, for which PSDs could be obtained at higher frequency resolution. This ruled out the possibility of potential EMG artifacts related to open eyes or poor frequency resolution in the eyes open data affecting our results. The results remained similar when we used a bipolar reference scheme instead of the original unipolar reference scheme. Further, although we observed a reduction in beta power in subjects with MCI or AD compared with their age and gender matched healthy controls, we did not find any significant change in slopes.

### Previous studies on PSD slope changes with age

Voytek and colleagues first showed the reduction in the slope of the PSD in older (60–70 years) participants compared with younger (20–30 years) in a visual working memory task (Voytek et al. 2015), which has now been confirmed in several studies involving a task paradigm (Dave et al. 2018; Tran et al. 2020; Ribeiro and Castelo-Branco 2022) as well as in resting state data (Hill et al. 2022; Merkin et al. 2023). However, all these studies dealt with frequencies less than 120 Hz, for example, 2–24 (Voytek et al. 2015), 2–20 (Tran et al. 2020), 1–40 (Hill et al. 2022), and 2–40 Hz (Merkin et al. 2023). In our data, PSDs were largely overlapping up to ~ 50 Hz (Fig. 1), potentially because our analysis was limited to the elderly population between 50 and 90 years, as opposed to a wider gap in age groups in other studies (e.g. 20–30 vs 60–70 in Voytek et al. 2015 and 18–35 vs 50–86 years in Merkin et al. 2023). We, therefore, chose a slightly higher frequency range of 64–140 Hz (LFR) in which the PSD flattening could be easily observed in the PSDs, and 230–430 Hz (HFR) in which steepening of PSD was easily noticed. One important point to note here is that the interstimulus baseline period during the “eyes open” state may not be the same as true “resting state” condition since the subjects were still engaged in a task.

#### Neural mechanisms underlying PSD slope changes

Although precise generative mechanisms underlying aperiodic activity is unknown, a multitude of factors have been suggested to describe the slopes, which include tissue properties (Bédard et al. 2006a), self-organized criticality (SOC; He et al. 2010; Chaudhuri et al. 2018), filtering properties of dendrites and extracellular medium (Bédard et al. 2006b; Logothetis et al. 2007; Bédard and Destexhe 2009), and excitation-inhibition (E/I) balance (Gao et al. 2017; Naskar et al. 2021). In addition, the PSD slopes depend on simple factors such as the reference scheme used for recording the data (Shirhatti et al. 2016).

Recently, a “neural noise” hypothesis has been proposed to explain the reduction in the PSD slope, which is related with increased asynchronous background neuronal firing (Voytek et al. 2015) or an increase in the E/I ratio (Gao et al. 2017). Specifically, Gao and colleagues developed a model in which Poisson distributed spikes arriving at excitatory and inhibitory neurons generated a change in excitatory and inhibitory conductance that were modeled as a difference-of-exponentials due to rise and decay time constants associated with AMPA and GABA_A_ receptors. Both these conductance profiles produced a low-pass effect, but slower kinetics of GABA_A_ caused a steeper roll-off with frequency compared with the AMPA conductance (see fig. 1 of Gao et al. 2017). Consequently, reduction in inhibition (increase in the E/I ratio), potentially due to dysfunctional inhibitory circuitry with aging, led to shallower slopes. This hypothesis has been supported by experiments in which the PSD slope was modulated by administration of drugs that led to either increased inhibition (e.g. propofol) or increased excitation (ketamine) (Gao et al. 2017; Lendner et al. 2020; Waschke et al. 2021). Our results could be consistent with this hypothesis, provided we modify the characteristics of this neural noise, which was band-pass as shown in Fig. 1(D).

Interestingly, surface EMG signals have energy between 40 and 200 Hz, peaking around ~ 100 Hz, which could explain at least part of the slope changes in the LFR (Muthukumaraswamy 2013; McManus et al. 2020). On the other hand, this cannot explain the effect in steepening of the slope in HFR, since the noise was similar whether eyes were open or closed and occurred at a higher frequency range than EMG activity. Indeed, the noise need not be myogenic but instead generated by the brain. As the timescales of fundamental neural processes like synaptic neurotransmitter diffusion time and timing of spike propagation lie below 10 ms (Sabatini and Regehr 1996; Shepherd 2004), high-frequency activity (>100 Hz) contains a wealth of information about neural processing and integration. Hence, the increase in slope in old group in HFR could be a reflection of age-related changes in these neural processes. In addition, it could be due to age-related alteration of physiological properties in brain tissue (Aalami et al. 2003; Lee and Kim 2022), cortical thickness (Lillie et al. 2016), and skin conductance (Lim et al. 1996; Venables and Mitchell 1996), which could determine the amplitude of this high-frequency signal measured from the scalp.

#### Variation in PSDs slopes with frequency and electrode location

The PSD slopes in our study varied considerably depending on the frequency range (Fig. 1), similar to what we observed in LFP data in a previous study (see fig. 1 of Shirhatti et al. 2016). We discuss some of these reasons below.

The initial high slope below 60 Hz as shown in Figs. 1–3, and Fig. 5 may be due to the presence of the oscillatory activity that may have persisted even after accounting for the oscillatory power using FOOOF. The periodic activity in theta and alpha frequency range has been shown to be correlated with the aperiodic activity computed in the similar frequency range (Donoghue et al. 2020a). Further, the oscillatory power and centre frequency of low-frequency oscillations also change with physiological aging (Voytek et al. 2015; Murty et al. 2020; Sahoo et al. 2020; Tran et al. 2020), which could further affect the slope. Indeed, researchers have stressed upon the need of removing the oscillations before computing the slopes indicating disruption of aperiodic activity in the LFRs (Merkin et al. 2023). At lower frequencies, the slopes are also heavily dependent on the reference scheme (Shirhatti et al. 2016). Hence, it may be better to perform slope analysis at frequencies which are devoid of oscillations. These low-frequency oscillations are unlikely to affect our main results since the difference in slopes with age mainly occurred beyond the frequency range dominated by oscillations (>64 Hz).

The dip in slope between 100 and 200 Hz and subsequent increase beyond 200 Hz reflects the presence of a “knee” in the PSD. This “knee” has been observed in previous studies as well, albeit at a different frequency. Miller and colleagues (Miller et al. 2009) found a knee in their ECoG PSD at ~ 75 Hz and attributed it to post-synaptic potential current of a particular timescale. In our data, the knee was present at ~ 175 Hz in all the electrode groups except the frontal group (where it was at ~ 100 Hz) and was prominent in occipital and temporal regions. This resulted in a timescale of ~ 5 ms, computed as 1/2$\pi{f}_{knee}$, ${f}_{knee}$ being the knee frequency. This timescale of ~ 5 ms may be due to post-synaptic current, tissue low pass filter (Miller et al. 2009), or characteristic initial adaptation period of pyramidal neurons (Rauch et al. 2003).

The surprising result of the dependence of the PSD slope on electrode location at high frequencies, which has been observed previously (Dehghani et al. 2010), could be due to inhomogeneity of skull thickness or conductivity (Law 1993; McCann et al. 2019). Future measurement of regional conductivity and its relation to PSD slope would be helpful to shed light on this issue.

#### PSD slope changes with neurological disorders

Previous studies have shown a reduction in low-frequency oscillatory activity as well as stimulus-induced gamma activity in MCI/AD subjects (Benwell et al. 2020; Murty et al. 2021; Ranasinghe et al. 2022), which we also found in our dataset. As AD is often associated with E/I imbalance (Lauterborn et al. 2021), and E/I ratio affects PSD slopes (Gao et al. 2017), we expected a change in PSD slope in MCI/AD subjects as well, but we found a null result. It could be due to a small sample size, although we found significant differences in power at both low-frequencies (Fig. 5) and in the gamma band (Murty et al. 2021) with the same sample size. Further, this null result is consistent with previously documented MEG observations (Benwell et al. 2020: 18 AD patients; Springer et al. 2022: 44 AD/MCI patients). This suggests that the relationship between PSD slope and E/I balance could be complicated and dependent on other factors, as discussed above. Nevertheless, a larger EEG dataset in future would be helpful to verify our results.

With the advancement of science, what was previously considered “noise” has been moved to the realm of “signal,” requiring an update of existing theoretical framework with new empirical results (Uddin 2020). For example, in functional magnetic resonance imaging (fMRI) measurements, the global signal (average value for whole brain signal) in the blood oxygen level dependent (BOLD) signal analysis which was considered “noise” and used to be removed, was later found to reveal important information with respect to individual behavior (Li et al. 2019) and psychopathology (Yang et al. 2017). Our present analysis, showing the age-related differences in the “noise considered” signal, is a step toward uncovering possible neurophysiological mechanisms hidden at these high frequencies in EEG. Detailed modeling and analysis in future is required to pinpoint the exact reasons for the peculiar age-related behavior observed in the present study.

In summary, along with the validation of previous signs of aging in aperiodic activity at low frequencies, our study sheds light on new hallmarks hidden at high frequencies. Our results, highlighting the age-related increment in slope in HFR, would help in bringing attention to the undergoing activity in the brain at high frequencies and unraveling its neural physiology. All electrodes exhibited the changes in aperiodic activity, indicating that the aperiodic activity reflects some global processes in brain, instead of being domain-specific. The indifference of slopes in AD patients would help in constraining the neurophysiological processes affecting AD and the aperiodic activity. Future research related to how HFR slope varies with anesthesia or other manipulations to change E/I balance would help in elucidating its underlying mechanisms, and would shed light on the neurobiology of aging in healthy and pathological brain.

## Supplementary Material

supplementary_material_tgad011

## Data Availability

All spectral analyses were performed using Chronux toolbox (version 2.10), available at http://chronux.org. Slopes were obtained using Matlab wrapper for FOOOF (https://github.com/fooof-tools/fooof_mat). Raw data will be made available to readers upon reasonable request and made publicly available at a later time.

## CRediT authors statement

Srishty Aggarwal (Formal analysis, Methodology, Writing—original draft, Writing—review and editing), Supratim Ray (Data curation, Funding acquisition, Methodology, Project administration, Resources, Supervision, Validation, Writing—original draft, Writing—review and editing).
