# Supplementary material for: Slope of the power spectral density flattens at low frequencies (<150 Hz) with healthy aging but also steepens at higher frequency (>200 Hz) in human electroencephalogram
Source: Cereb Cortex Commun. 2023 Jun 6;4(2):tgad011. doi: 10.1093/texcom/tgad011 (PMC10276190; doi:10.1093/texcom/tgad011)
Supplement: supplementary_material_tgad011 [file supplementary_material_tgad011.pdf]

Supplementary Material

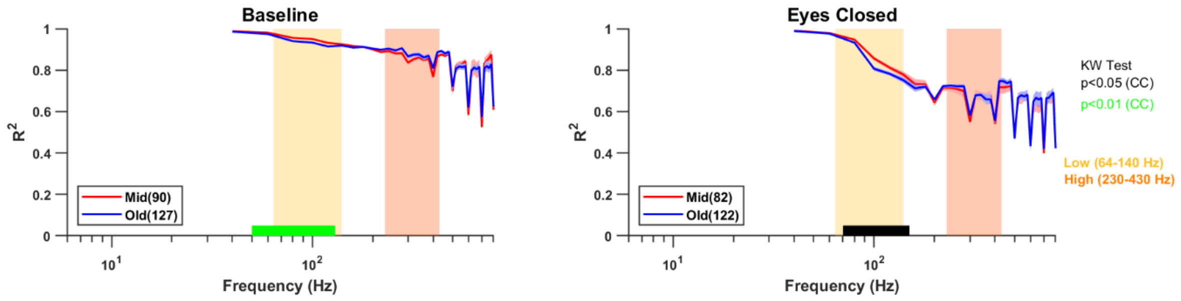

Supplementary Figure 1. R^2 for all electrodes for eyes open (left panel) and eyes closed (right panel) state. Solid traces represent the median for mid and old age groups respectively and shaded region around them indicates ±SEM across subjects, computed after bootstrapping over 10,000 iterations. The numbers in legend in the top panel represent the subjects in the respective age groups. Horizontal bars at the abscissa in the bottom panel represent significance of differences of slopes between mid and old (black: p<0.05 and green: p<0.01, K-W test, Cluster Corrected (CC)). The abrupt reduction in R^2 at frequencies in multiple of 100 Hz may be due to poor fitting in presence of line noise at the edges of fitting interval.

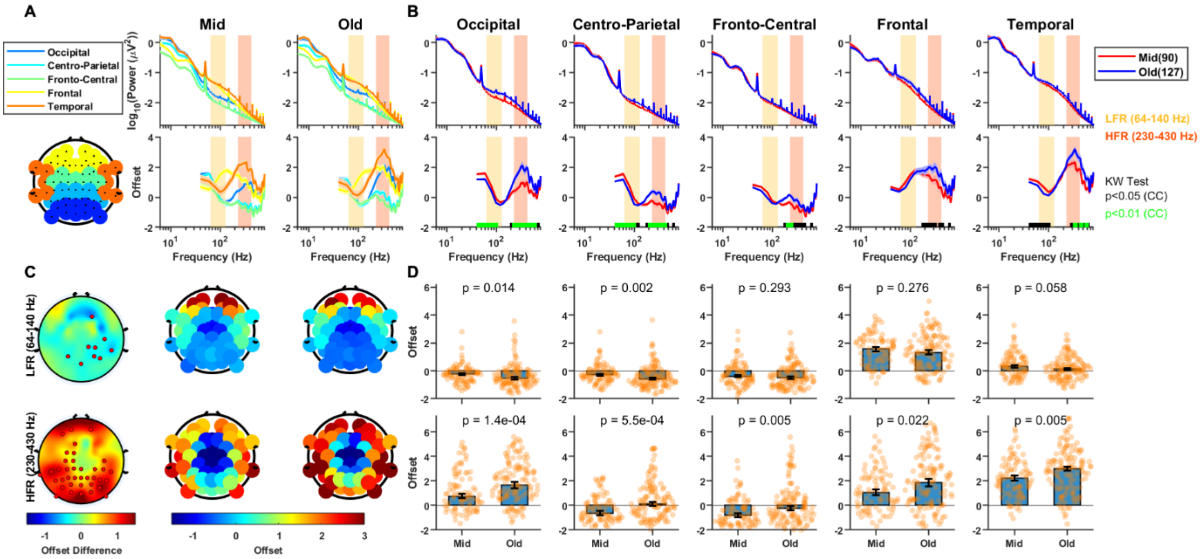

Supplementary Figure 2. Variation of PSDs and offset with frequency across the two age groups in various areas of brain for eyes open state. Same as Figure 2 but for offset instead of the slope.
